# Complete Genome Sequence of an *mcr-10-*Possessing Enterobacter roggenkampii Strain Isolated from a Dog in Japan

**DOI:** 10.1128/MRA.00426-21

**Published:** 2021-07-29

**Authors:** Toyotaka Sato, Masaru Usui, Kazuki Harada, Yukari Fukushima, Chie Nakajima, Yasuhiko Suzuki, Shin-ichi Yokota

**Affiliations:** a Department of Microbiology, Sapporo Medical University School of Medicine, Sapporo, Japan; b Laboratory of Food Microbiology and Food Safety, Department of Health and Environmental Sciences, School of Veterinary Medicine, Rakuno Gakuen University, Ebetsu, Japan; c Department of Veterinary Internal Medicine, Tottori University, Tottori, Japan; d Division of Bioresources, Hokkaido University, Research Center for Zoonosis Control, Sapporo, Japan; e International Collaboration Unit, Research Center for Zoonosis Control, Hokkaido University, Sapporo, Japan; Indiana University, Bloomington

## Abstract

The complete genome sequence of *mcr-10*-possessing Enterobacter roggenkampii En37, isolated from a dog in Japan, was determined. *mcr-10* was located on a 70,277-bp IncFIB plasmid without any additional antimicrobial resistance genes.

## ANNOUNCEMENT

Colistin is used as a last-line antimicrobial against multidrug-resistant Gram-negative bacteria, including Enterobacter cloacae complex. Therefore, colistin resistance has become a therapeutic problem. The plasmid-mediated colistin resistance gene, *mcr*, encodes phosphoethanolamine transferase, which reduces the colistin susceptibility by modifying lipid A of lipopolysaccharides with phosphoethanolamine (1). The *mcr* variants from *mcr-1* to *mcr-10* have been reported worldwide, whereas few have been reported for Mcr-10 (2).

We isolated a colistin-resistant Enterobacter roggenkampii strain, En37, from pus of a dog in 2015 in Japan without ethical approval, according to the Japanese government guidelines (3). En37 was subcultured on CHROMagar ECC (Becton, Dickinson, NJ) at 37°C overnight and proceeded to the genomic DNA isolation. The MIC(s) of colistin was 16 or 32 mg/liter according to Clinical and Laboratory Standards Institute guidelines (4). The genomic DNA for short- and long-read sequencing was extracted using a Wizard genomic DNA purification kit (Promega, Madison, WI) and Genomic-tip 20/G and Genomic DNA buffer set (Qiagen, Hilden, Germany), respectively.

Short-read sequencing data were obtained by Nextera XT and MiSeq sequencing (Illumina, San Diego, CA) with 300-bp paired-end reads according to the manufacturer’s protocol. The resulting reads (length, 1,417,426,773 bp; number, 627,010) were trimmed by using fastp v0.20.1 (5). FastQC v0.11.9 was used for quality control.

Long-read sequencing data were obtained with rapid barcoding sequencing kit SQK-RBK004, and MinION sequencing data were obtained with a FLO-MIN-106 R9.4 flow cell (Oxford Nanopore Technologies, Oxford, UK) according to the manufacturer’s protocol. All bead washing steps were performed using AMPure XP beads (Beckman Coulter, Brea, CA). The base calling was performed on MinKNOW software (Oxford Nanopore Technologies) for the full 48-h run time with no alterations to any voltage scripts. The obtained 627,010 reads (*N*_50_, 10,579 bp) were demultiplexed, and adaptors were trimmed using Porechop v0.2.4 (https://github.com/rrwick/Porechop) and then quality-filtered using Nanofilt (Q score, 10; minimum length, 1,000 bp) (6). NanoStat v1.5.0 was used for quality control and error correction.

Long-read sequencing reads were error-corrected using short-read sequencing reads with LoRDEC v0.6 (7). *De novo* assembly of error-corrected long-read sequencing reads was performed using Flye v2.8 (8). Assembled contigs were error-corrected using short-read sequencing reads with Pilon v1.24 (9). Finally, the assembled sequences were annotated using DFAST v1.1.0 with standard settings (10). Default parameters were used for all software unless otherwise indicated.

We obtained three different complete genome sequences (Table 1). *mcr-10* was located on the IncFIB plasmid, namely, pEN37S. There were no additional antimicrobial resistance genes on pEN37S, as revealed by ResFinder 4.1 (11). pEN37S possesses 99.9% similarity with an *mcr-10*-carrying plasmid of *E. roggenkampii* strain Ecl_20_981 derived from medical wastewater in China in 2019 (GenBank accession number CP048651.1), according to a BLAST search. Therefore, *mcr-10* has been disseminated among *E. roggenkampii* in several countries.

**TABLE 1:** Features of En37 genomes

En37 genome	Name	Length (bp)	Coverage (×)	G+C content (%)
Chromosome	En37 chromosome	4,846,950	185	55.9
Large plasmid	pEN37L	144,031	269	53.5
Small plasmid	pEN37S	70,277	488	52.4

### Data availability.

The whole-genome sequence was deposited at DDBJ/ENA/GenBank under the accession numbers AP024495 (chromosome), AP024497 (pEN37S), and AP024496 (pEN37L). The Illumina and MinION sequence reads were deposited in the Sequence Read Archive (SRA) database under accession numbers DRR285169 and DRR285170, respectively.
